# Nontraumatic liver herniation mimicking a right lower lobe lung mass

**DOI:** 10.1093/jscr/rjab387

**Published:** 2021-09-14

**Authors:** Muhammad Adeel Samad, Annis Ali, Diane C Shih-Della Penna, Scott Tiedebohl

**Affiliations:** WellSpan York Hospital, Department of Surgery, York, PA, USA; WellSpan York Hospital, Department of Surgery, York, PA, USA; WellSpan York Hospital, Department of Surgery, York, PA, USA; WellSpan York Hospital, Department of Surgery, York, PA, USA

## Abstract

Nontraumatic liver herniation through diaphragm is a rare condition. We present a case of a 54-year-old female presenting with nontraumatic liver herniation mimicking a right lower lobe mass. Patient was noted to have growth of two right lower lobe lung nodules from 1.5 cm × 2.8 cm and 0.9 cm × 1.3 in August 2009 to 2.8 cm × 4.1 cm and 1.1 cm × 1.4 cm in March 2019 on computerized tomography (CT) scan. PET scan as well as the growth pattern was consistent with low-grade malignancy likely carcinoid tumor. CT-guided biopsy was not feasible because of location of the mass. We performed robotic thoracoscopy with plan for wedge resection, however gross inspection of the thoracic cavity revealed two masses on the dome of the diaphragm with appearance like liver and correlating with nodules seen on CT scan. A core needle biopsy showed that it was benign liver tissue.

## INTRODUCTION

Spontaneous diaphragmatic herniation is a rare type of acquired diaphragmatic hernia without any history of trauma [1]. We describe a case where a nontraumatic liver herniation through the diaphragm was observed mimicking a right lower lobe lung mass.

## CASE REPORT

**Figure 1:**
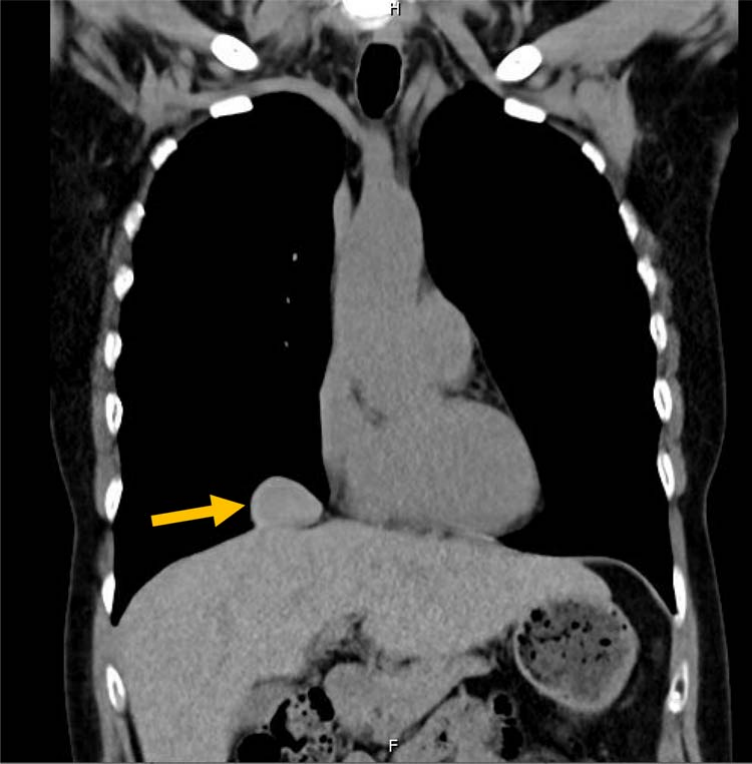
Larger lung nodule measuring 1.5 cm × 2.8 cm on CT scan.

**Figure 2:**
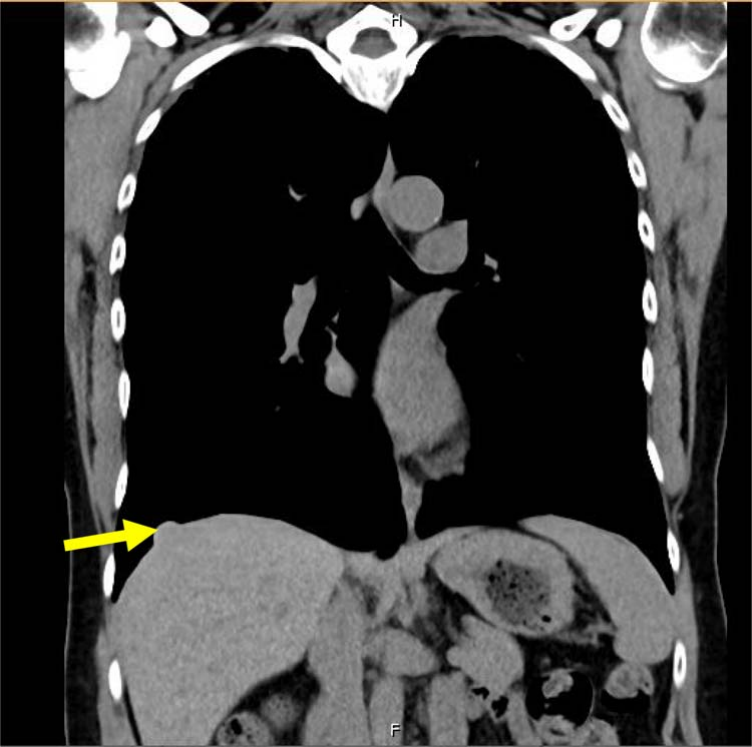
Smaller nodule measuring 0.9 cm × 1.5 cm on CT scan.

**Figure 3:**
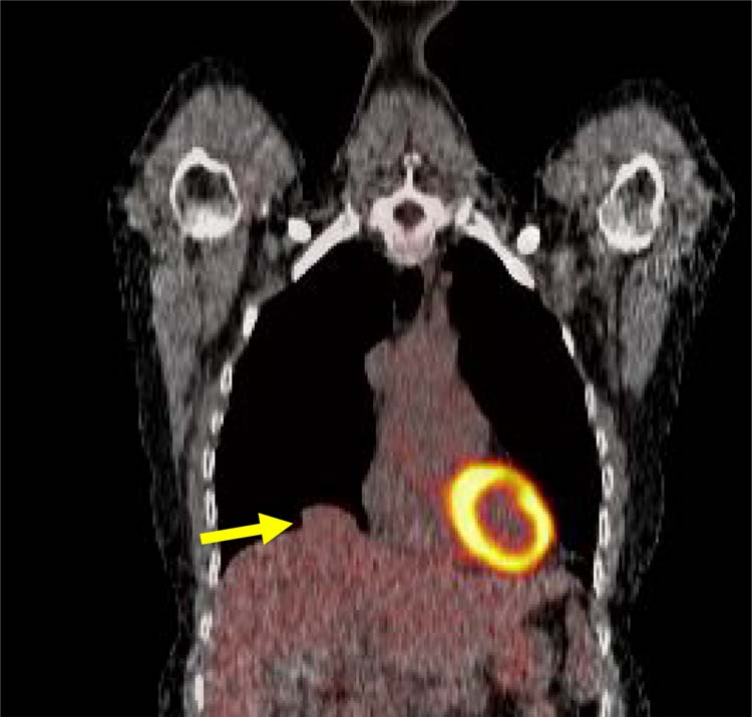
Larger nodule on PET scan

**Figures 4:**
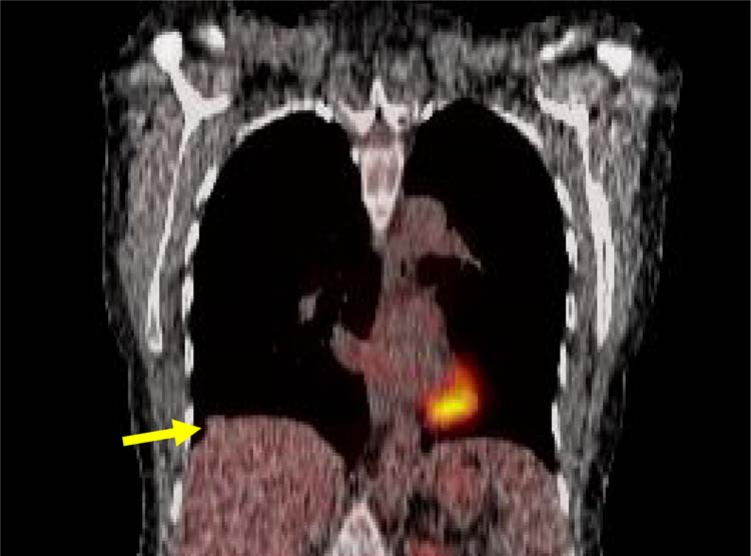
Smaller nodule on PET scan.

A 54-year-old female without prior thoraco-abdominal trauma presented to the office with two right lower lobe lung nodules. The nodules were initially noted incidentally on a computerized tomography (CT) scan in August 2009 and measured 1.5 cm × 2.8 cm and 0.9 cm × 1.3 cm (Figs 1 and 2). A follow-up CT scan and positron emission tomography (PET) performed in 2010 demonstrated that the nodules were stable in size. The patient was lost to follow-up until March 2019 when she had a CT scan for concern for pneumonia. On these images, the lung nodules had increased in size to 2.8 cm × 4.1 cm and 1.1 cm × 1.4 cm. The patient was otherwise asymptomatic. A PET scan was obtained which showed hypermetabolic nodules with maximum SUV of 3.29 and 1.4, for the larger and smaller nodule, respectively (Figs 3 and 4). Radiographic appearance as well as the growth pattern was consistent with low-grade malignancy suspicious for carcinoid tumor. CT-guided biopsy was not feasible because of location of the tumor.

**Figure 5:**
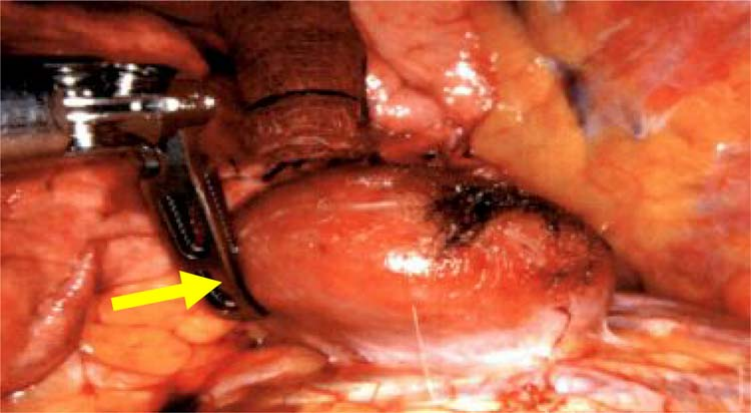
Liver herniation corresponding to the location of larger lung nodule seen on CT scan.

**Figures 6:**
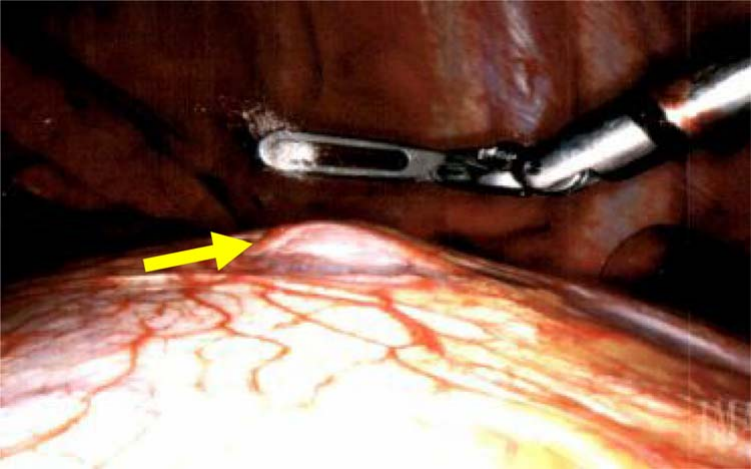
Liver herniation corresponding to the location of smaller lung nodule seen on CT scan.

We performed a robotic thoracoscopy with plan for wedge resection and possible completion lobectomy. Gross inspection of the thoracic cavity revealed an abnormal mass on the dome of the diaphragm with the appearance similar to liver (Figs 5 and 6). The mass was noted to be positioned in the oblique fissure and correlated with the location of the larger nodule seen on CT scan. There was a second area of thinned-out diaphragm where a small nodule was noted to be protruding under the diaphragm. This second mass also had the appearance of liver and was consistent with location of smaller lesion on CT scan. A core needle biopsy was performed which was consistent with benign liver tissue. The decision was made not to proceed with any diaphragmatic repair or reinforcement with mesh. This was diagnosed as an asymptomatic herniation through the diaphragm, and the liver was fused to the diaphragm therefore preventing future intestinal herniation.

## DISCUSSION

Nontraumatic herniation of the liver through diaphragm is a rare phenomenon. A review of literature (1956–2009) by Losanoff *et al*. [2] revealed that there are 28 reported cases of spontaneous diaphragmatic rupture (SDH) and of those only 10% had liver herniation.

SDH is caused by events that increase intra-abdominal pressure like complicated labor, intense physical exercise, psychiatric illness and cough secondary to pertussis [3]. Another predisposing factor for SDH is a congenital diaphragmatic defect which creates an area of weakness and increases the likelihood of herniation due to excess intra-abdominal pressure. These defects are formed due to lack of fusion of diaphragm during embryonic development. Posterolateral defect (Bochdalek hernia, 95%) is the most common type, followed by anterior-retrosternal defects (Hernia of Morgagni, 4%) and hiatal hernias and septum transversum defects (1%) [4]. It is unclear if the etiology of SDH is the lack of muscular coordination during intense activity, preexisting diaphragmatic defect creating a weak spot, or both [1].

SDH has also been described in association with other conditions like Ehler-Danlos Syndrome (EDS) and endometriosis because of weakness of diaphragmatic tissue. EDS is a hereditary disorder that leads to abnormalities in the synthesis and structure of collagen. This leads to tissue fragility and weakness, and incidence of diaphragmatic herniation has been noted up to 22% [5]. In patients with endometriosis, ectopic endometrial nodules on diaphragm undergo cyclic necrosis leading to diaphragmatic fenestrations. These can coalesce into a large defect and eventually lead to herniation of abdominal viscera into the thoracic cavity [6]. Ilyas *et al*. [7] described a case of patient with history of endometriosis who was noted to have mass within the right pleural space on CT scan. Intraoperatively, the patient was noted to have liver herniation which was mimicking a right lung mass on CT scan. Our patient did not have history of endometriosis.

To the best of our knowledge, there are only two reported cases of nontraumatic liver herniation in current literature in absence of congenital diaphragmatic defects and medical conditions like EDS and endometriosis. Loumiotis *et al*. [1] reported a patient who presented with acute substernal pain after attempting to lift a heavy object and was found to have herniation of liver on CT scan. Patient underwent exploratory laparotomy and diaphragmatic defect was between the retrohepatic inferior vena cava (IVC) and the right crus of the diaphragm. The caudate lobe of liver was reduced and hernia was repaired with 2-0 Prolene [1]. Luo *et al.* [8] presented a patient with no prior history of thoracoabdominal trauma who was found to have right lower lobe mass on chest X-ray and CT scan. Intraoperatively, the mass was identified as a nontraumatic liver herniation encased in a thin sac formed by diaphragm. Resection was performed and it was noted to be liver on final pathology [8]. Our case is unique given that the patient had an asymptomatic herniation presenting as a lung mass without any prior history of trauma or risk factors for increased intraabdominal pressure. She did not have any history of endometriosis or EDS. Our decision to forego surgical repair was based on the facts that patient was asymptomatic and the liver was fibrosed to the diaphragm preventing herniation of other abdominal contents.

## DECLARATIONS

Ethics approval and consent to participate: IRB has determined this project does not meet the definition of human subject research.
